# The prevalence of iron deficiency anemia among African asylum seeking children residing in Tel Aviv

**DOI:** 10.1186/s13584-019-0351-3

**Published:** 2019-11-25

**Authors:** Gideon Koren, Lielle Ross, Oren Zwang, Orel Benari

**Affiliations:** 1 Terem Refugee Clinic, Tel Aviv, Motherisk Program, Shamir Hospital and Adelson Faculty of Medicine, Ariel University, Ariel, Israel; 2 Motherisk Program, Shamir Hospital, and Adelson Faculty of Medicine, Ariel University, Ariel, Israel; 3 Adelson Faculty of Medicine, Ariel University, Ariel, Israel

**Keywords:** Anemia, African asylum seekers, Eritrea, Sudan, Iron deficient anemia, Children, Refugee children, Iron deficiency anemia, Refugees, Africa, Children

## Abstract

**Background:**

It has been the impression of pediatricians at the Terem Clinic for African asylum seekers in Tel Aviv that they encounter large numbers of anemic children.

The objectives of this study were 1) to quantify the prevalence of anemia among African African asylum seeking children treated in the Terem Clinic for refugees in Tel Aviv; 2) to compare it to the rates among Jewish Israeli children; 3) and to correlate it with their nutritional iron intake. Overall, this effort aims at informing changes in policies and practices that will ensure healthy development of African asylum seeking children in Israel.

**Methods:**

The prevalence of anemia was calculated for all toddlers and children under the age of twelve years visiting the refugee clinic and compared to the recently reported rates of anemia among urban Jewish Israeli children of similar ages; Nutritional iron intake was calculated in a subgroup by a food frequency questionnaire translated to Amharic and Tigrinya.

**Results:**

Mean age of the children (SD) was 2.96 yr. (SD 2.77) and mean hemoglobin 10.88 g/dl (1.47). Out of 386 eligible children, 131(34%) were anemic, fourfold more prevalent than reported among 263 Jewish toddlers and young children of the same age group [(11%), OR 4.15(95% ci 2.67–6.43)].

In a subgroup (*n* = 26) investigated for amount of daily iron intake, 46.2% did not receive the recommended daily allowance for their age. Nine of them had received iron supplements.

**Conclusions:**

Low hemoglobin levels are four-fold more prevalent among the African asylum seeking children. The dietary data suggest iron deficiency as a major cause, although other etiologies need to be ruled out. Because of the adverse long term impact of early anemia on child development, new policies need to be developed to ensure that refugee children develop in a healthy manner. These should include routine mandatory supplements of iron for all refugee children, in parallel to developing an educational program for parents how to achieve iron-sufficient diets for their children. Further research is needed to guide public health action for these children.

## Introduction

Iron deficiency anemia is the most prevalent form of anemia around the world, adversely affecting large numbers of young children. Children of low socioeconomic class or of refugee status are particularly at risk due to their attendant low intake of iron –rich food [1]. The issue pertaining to refugee and asylum seeking children has been recognized around the world. In the Middle East, among Syrian refugee children in a tertiary hospital which may be confounded by serious illnesses,, anemia was present in 50% of participants [2], similar to the rate of 48.4% at the Za’atri camp in Jordan [3]. Among Gaza Strip Palestinian refugee children the overall prevalence of anemia was 59.7%, higher among poor households, and correlating with underweight [4].

Since the early 1990s increasing numbers of African asylum seekers have immigrated to Israel from Eritrea (80%) and Sudan (20%) [5], and by April 2018 their numbers exceeded 45,000, not including the children born in Israel [6].

Based on preliminary chart reviews, it has been the impression of pediatricians attending the Terem Clinic for refugees in Tel Aviv, that incidentally large numbers of the children attending the clinic exhibit anemia. This presents a critical public health issue, as iron deficiency anemia has been repeatedly shown to be associated with long term cognitive developmental deficits [7–9]. Due to the fact that minority, under privileged children are at increased developmental risks from numerous other risk factors [10], such adverse long term effects of anemia should be addressed rigorously.

The objectives of the present study were 1) to quantify the prevalence of anemia among African African asylum seeking children treated at the Terem Clinic for asylum seekers in Tel Aviv; 2) to compare it to the reported prevalence of anemia among urban Jewish Israeli children of similar ages; and 3) to estimate their nutritional iron intake.

The overall aim of these inquiries is to develop an improved strategy for addressing this serious public health issue.

## Patients and methods

### Setting

The study was approved by Assuta hospital Research Ethics Board in Tel Aviv. The Terem Clinic for refugees, located in the Central Bus Station in Southern Tel Aviv, serves the medical needs of refugees who are not insured in any of the four health maintenance organizations operating In Israel. Funded by the Ministry of Health, the clinic relies for much of its work on physicians volunteering to attend the clinic.

Included in the present study were all children 9 months to 12 years of age attending the clinic between January 1 and June 30, 2018. For the purpose of this study we retrieved all CBC measurements performed on children as part of the diagnostic workup of fever or trauma without blood loss, but not of anemia, weakness or related complaints. The rational for this decision was to avoid bias of over representation of children with anemia, in order to better reflect the true prevalence of anemia among these children. Hemoglobin was measured by Coulter STKS (Coulter Corporation, Hialeah, FL, USA).

Using Chi square test, we compared the prevalence of anemia among the asylum seekingchildren to the prevalence published recently by Moshe et al. for healthy Israeli Jewish children of the same age group in Jerusalem [11]. The rational was to facilitate comparison of the refugee children to Jewish children of similar age range, also residing in an urban setting of the same country, and hence, to similar nutritional iron sources.

In a subgroup focused analysis we recruited 26 young children who had a CBC taken as part of their medical workup. With informed parental consent we inquired about the child’s diet using a food frequency questionnaire delivered to the parents in Tigrine and Sudanese. To account for food consumed by the children in day-care and kindergarten, we further collected the meal plan from Unitaf – an institution that provides day care and after school programs for children with no Israeli status, and which the majority of refugee children in Tel Aviv attend. For the focus group cases we retrieved the child’s age, body weight, height, and levels of hemoglobin, hematocrit, mean corpuscular volume, mean corpuscular hemoglobin, and mean corpuscular hemoglobin concentrations. With the use of an abbreviated food questionnaire [12] translated to Amharic and Tigrinya, we calculated the children’s daily nutritional iron intake. We calculated the proportion of children receiving iron below the recommended daily allowance of 11 mg for 7–12 months of age, 7 mg for ages 1–3 years, and 10 mg between 4 and 8 years [13].

## Results

Between January 1 and June 30, 2018 there were a total of 4028 pediatric visits to the clinic, and in 386 of them CBC was performed for causes other than anemia. Out of these, 131 children (34%) had hemoglobin levels below 11 g/dL. This rate was fourfold higher than among 29 of 263 toddlers and young Jewish Israeli children (11.2%) [8], (OR 4.15(95% confidence intervals 2.67–6.43) (*p* < 0.0001) (Table 1).

**Table 1:** Characteristics of study population

Period:	Jan 1-June 30, 2018
Total pediatric visits:	4028
Total CBC measures	440
Children meeting inclusion criteria	386
Prevalence of anemia	131 (34%)

In the subgroup analysis studied for iron intake, there were 26 children. Eleven of them (42.3%) had hemoglobin < 11 g/dL. The mean daily nutritional iron intake was 8.4 mg (SD 0.47). All 26 (100%) had low hematocrit (< 41%), 21(80.8%) had low mean corpuscular volume (> 78 fL), and 18(69.2%) had low mean corpuscular hemoglobin (< 27 pg). Eleven of them had a daily nutritional iron intake lower than recommended daily allowance for their age by the Canadian Paediatric Society. Nine of them were prescribed iron supplement (Table 2).

**Table 2:** Hematological characteristics among 26 African asylum seeking children where nutritional iron intake was calculated

	Age (yr)	Wt(k	Hgb	Hct	MCV	MCH	MCHC	Nutritional iron
1	2.4	14	11.3	31.4	75.2	27.1	36	5.5
2	3	16	11.7	32.8	74.8	26.7	35.6	7.05
3	1.75	12	7.1	21.9	53.6	17.4	32.4	7.05
4	1.1	10	11	32.4	63.5	21.5	33.9	9.75
5	2	16	11.6	33.3	75.6	26.4	34.9	9.3
6	5.4	21	11.9	33.4	80.7	28.7	35.6	3.1
7	0.83	9.1	10.8	31.4	69.7	24	34.4	5.0
8	1.33	10.1	10.3	29.6	73.8	25.7	34.8	6.8
9	1.4	11.5	8.8	27	57	18.6	32.6	2.95
10	1.15	10.1	11.3	32.7	68.8	23.8	34.6	6.65
11	0.9	10	11.2	32	74.5	26.1	35	7.65
12	7	29	12.7	36.5	81.4	28.3	34.8	8.25
13	3.7	15	12.1	34.3	80.3	28.3	35.3	10.1
14	2	11.5	12.3	35.2	73.7	25.7	34.9	13.6
15	0.75	10.1	11.6	33.4	71.9	24.9	34.7	4.47
16	2.8	13.4	11.8	33.8	73.9	25.8	34.9	15.7
17	10.8	37	9.3	28.2	74.3	24.5	33	13.95
18	3	14	10.5	29.6	76.5	27.1	35.5	4.52
19	11.15	33	11.6	33.5	82.8	28.7	34.7	13.8
20	3.5	14.5	12.4	35.8	76.6	26.6	34.7	12.77
21	1.4	13.6	10.4	31	67.6	27.7	33.5	9.07
22	1.9	12.2	10.2	29.4	74.9	26	34.7	6.97
23	3.5	13	11.3	32.1	74.9	26.3	35.2	9.02
24	1.1	9.8	12	34.8	7 7.7	26.8	34.5	9.05
25	1.8	16.5	10.9	31.7	81.2	27.9	34.3	6.95
26	1.4	12.5	6.8	19.8	74.4	25.6	34.4	8.4

## Discussion

The major causes of the global burden of anemia worldwide are iron deficiency, malaria and parasitic infections[14]. There is ample evidence that refugee and asylum seeking children worldwide suffer from iron deficiency anemia. A combination of low birth weight, low intake of iron-rich foods, low socioeconomic status, and parents’ lack of familiarity with appropriate nutritional needs synergize each other [1, 15, 16]. The existing evidence of permanent long term developmental delays after iron deficiency anemia in early childhood, even if subsequently corrected [8], emphasizes the need for proactive, well organized programs for early prevention. Even in developed countries, where programs for nutrition assistance exist, up to one-third of refugee children are not enrolled in such programs [16].

The aim of the present study was to objectively substantiate impressions that African asylum seeking children in Tel Aviv suffer from high prevalence of iron deficient anemia.

The results of this study confirm the impressions of pediatricians attending the refugee clinic, suggesting that the African refugee children treated at the Terem clinic exhibit a fourfold increased risk for microcytic anemia as compared to Israeli Jewish children of similar ages in Jerusalem [11]. In an additional cross-sectional retrospective study in 34,512 insured Israeli infants aged 9 to 18 months, after excluding children with abnormal white or with chronic diseases, the prevalence of anemia was 15.5%. The prevalence was significantly higher in the non-Jewish population (22.5%) [17].

Our research provides evidence of iron intake below the recommended daily allowances in the majority of children we questioned. While in Israel there is a strong recommendation to supplement iron to young children, none of the studied children received it prophylactically, probably because they were not members of any of the 4 existing HMOs where such initiatives are closely adhered to. The typical Eritrean and Sudanese diets are not iron-rich; cooked beef, veal, sardines, chicken, ham, shrimp, oysters and dark leafy greens were not part of the diets of most of the surveyed children (data not shown) Our study population included children examined for reasons other than anemia. Infections in children have not been shown to induce iron deficiency anemia, however while this is true regarding MCV, MCHC, hemoglobin may drop in children with acute infection [18], and infections can confound certain iron markers, such as elevation in serum ferritin [19].

Potential limitations of our study need to be addressed: We could include only the subset of African asylum seeking children for whom CBC measurements were performed. It could be argued that this subset of children may not represent all asylum seeking children. However, because we excluded children for who CBC was performed for anemia, weakness, and because the common denominator of most asylum seeking children is lack of medical insurance, it is very unlikely that these data are not generalizable. Moreover, the Terem facility is the only place attending to the medical needs of these children in Tel Aviv, hence it is unlikely that there is a referral bias.

This study is dealing with the lowest socioeconomic status among the children of the asylum seekers. They are compared to Jewish children of Jerusalem. Thus, from a methodological point of view one can foresee that they will be more anemic. Clearly low SES is a big component in being asylum seekers. Of interest, Jerusalem, where our reference study came from is among the lowest overall SES in the nation, and still the Terem children are thrice more likely to present with anemia.

Interviews with the parents in our study have revealed lack of knowledge about the importance of prevention of iron deficient anemia, as well as the importance of balanced diets to ensure the wellbeing of their children. In general, the refugee children do not receive sufficient amounts of iron-rich nutrients such as beef, veal, sardines, chicken, ham, shrimp, oysters or dark leafy greens, and are exposed mostly to carbohydrates.

In terms of health policy, we believe that the way to correct this unacceptable situation should include a two prone approach:

1. Mandatory iron supplementation to all young asylum seeking children after a baseline CBC is obtained. The Ministry of Health of Israel presently recommends routine iron supplements to infants up to the age of 18 months. The Israeli Pediatric Society further states that usage of iron products should aim at prevention of anemia, and not products defined as food supplements [20], however, these recommendations do not seem to be widely executed among African asylum seeking toddlers. This may reflect the fact that many of them do not have medical insurance and, hence, are not regularly cared by a pediatrician or family physician.
2. Establishing an educational program for the parents. These should include a written pamphlet in Tigrine that summarizes in understandable terms the nutritional needs of the child and the feeding practices that can achieve them. In parallel, meeting of the parents with a nutritionist, either for all children, or focusing on anemic children, may facilitate long- lasting effects for the children, as well as for their siblings. Because many of these young children are not regularly attended by a physician, the parents need also to know that their young children are entitled to and should receive iron supplements as a medicinal preparation [20].
  It is acknowledged that other nutritional needs are not met in these children for the same reasons they do not receive appropriate iron supplements. Further studies are needed to guide similar public health policy in these children to address other potential nutritional deficiencies such as B12, calcium and other micronutrients. In view of our present findings, such studies are now planned.

## Conclusions

The proposed health policy strategy to address these issues includes supplementary iron for all young African asylum seekingchildren attending the clinic, as well as educational programs for the parents in their mother tongues to ensure maximal effectiveness. Because of the adverse long term impact of early anemia on child development, supplements should be given, and parents should be educated about iron-sufficient diets for their children.

## Data Availability

All relevant data appears in the manuscript and table.

## Abbreviations

CBC: Complete blood count
